# Therapeutic efficacy and safety of PCSK9-monoclonal antibodies on familial hypercholesterolemia and statin-intolerant patients: A meta-analysis of 15 randomized controlled trials

**DOI:** 10.1038/s41598-017-00316-3

**Published:** 2017-03-22

**Authors:** Li Jun Qian, Yao Gao, Yan Mei Zhang, Ming Chu, Jing Yao, Di Xu

**Affiliations:** 1 Department of Geriatrics, First Affiliated Hospital of Nanjing Medical University, Nanjing, 210029 China; 2 Department of Cardiology, First Affiliated Hospital of Nanjing Medical University, Nanjing, 210029 China

## Abstract

Proprotein convertase subtilisin/kexin9 monoclonal antibodies (PCSK9-mAb) have been studied intensively to identify their effect in lowering levels of low density lipoprotein cholesterol (LDL-C). However, the applicable target of PCSK9-mAbs remains inconclusive so far. Therefore, this first meta-analysis was carried out to clarify the therapeutic efficacy and safety of PCSK9-mAbs on the potential patients: familial hypercholesterolemia and statin-intolerant patients. All randomized controlled trials that met the search terms were retrieved in multiple databases. Efficacy outcomes included parameter changes from baseline in LDL-C and other lipid levels. Therapeutic safety were evaluated by rates of common adverse events. A total of 15 studies encompassing 4,288 patients with at least 8 weeks duration were selected. Overall, the therapeutic efficacy was achieved with significant reduction in LDL-C, TC, TG, Lp(a), Apo-B versus placebo. The decline in familial hypercholesterolemia patients (−53.28%, 95% CI: −59.88 to −46.68%) was even more obvious than that in statin-intolerant patients (−34.95%, 95% CI: −41.46 to −28.45%). No obvious safety difference was found out in the rates of common and serious adverse events. To conclude, PCSK9-mAb contributes to the decreased level of LDL-C and other lipids in familial hypercholesterolemia and statin-intolerant patients with satisfactory safety and tolerability.

## Introduction

Familial hypercholesterolemia (FH) and statin-intolerant patients are suffering high risk of cardiovascular disease (CVD)^1, 2^. Clinical guidelines advocate that low density lipoprotein cholesterol (LDL-C) is the target of CVD’s primary or secondary prevention^3^. Statins, the recommended first-line therapy to control lipidemia, tend not to be thoroughly effective^4^. In addition, of approximately 20 million statin users, an estimated 10% to 20% are statin-intolerant^5^, failing the goals of lowering blood lipid profile or developing statin-intolerance, such as injection-site reaction, nasopharyngitis, upper respiratory tract infections, influenza, cough, nausea, myalgia, myositis, headache, diarrhea, fatigue, abnormal pain, rectal bleeding, dehydration, arthralgia, back pain etc.^6–17^ FH, one of the most common genetic disorders in humans that endangers approximately 12 million people worldwide, can elevate the significant premature CVD morbidity and mortality^18^. For extremely high baseline LDL-C levels of FH, usually >5.2 mmol/L (200 mg/dL), even statins cannot achieve intensive LDL-C lowering targets, leading to early CVD with typical onset before age 50 in men and 60 in women^19^.

Proprotein convertase subtilisin/kexin9 (PCSK9) monoclonal antibodies, as an attractive therapy for lowering LDL-C levels^20^, can bind the LDL-receptor (LDL-R) on the surface of hepatocytes, interfering with LDL clearance in circulation, hence playing a pivotal role in regulating cholesterol homeostasis^21^. The human monoclonal antibodies against PCSK9 primarily include AMG145/Evolocumab and REGN727/SAR236553/Alirocumab, while RN316/bococizumab, RG7652, LY3015014, and ALN-PCSSC^22–25^, are now genetically validated as novel PCSK9-mAb therapies.

In the last 4 years, early clinical trials have proven that PCSK9-mAb can lower the plasma LDL-C level in FH and statin-intolerant patients. The other lipids and lipoproteins; high density lipoprotein cholesterol (HDL-C), total cholesterol (TC), triglycerides (TG), lipoprotein(a) (Lp(a)), apolipoprotein-B (Apo-B) and apolipoprotein-A1 (Apo-A1) can also benefit. Currently, there is no report to comprehensively pinpoint the applicable targets of PCSK9-mAbs—FH and statin-intolerant patients with sufficient clinical outcomes. To compare the efficacy and safety of PCSK9-mAb on FH and statin-intolerant patients, a total of 15 articles were assessed in this meta-analysis.

## Results

### Study selection and characteristic

**Figure 1:**
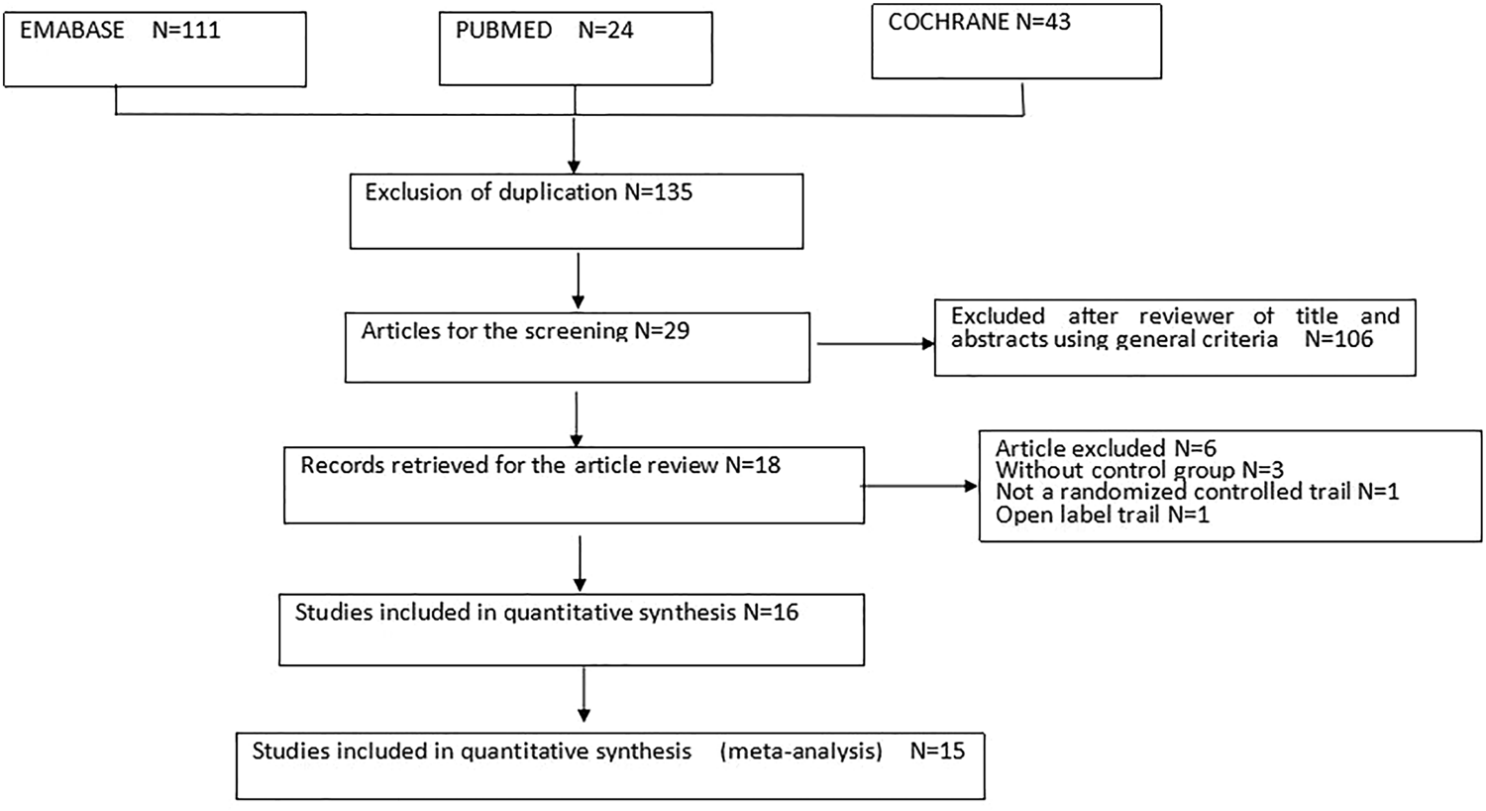
Preferred reporting items for systematic review and meta-analysis (PRISMA) flowchart of the process of study selection.

**Figure 2:**
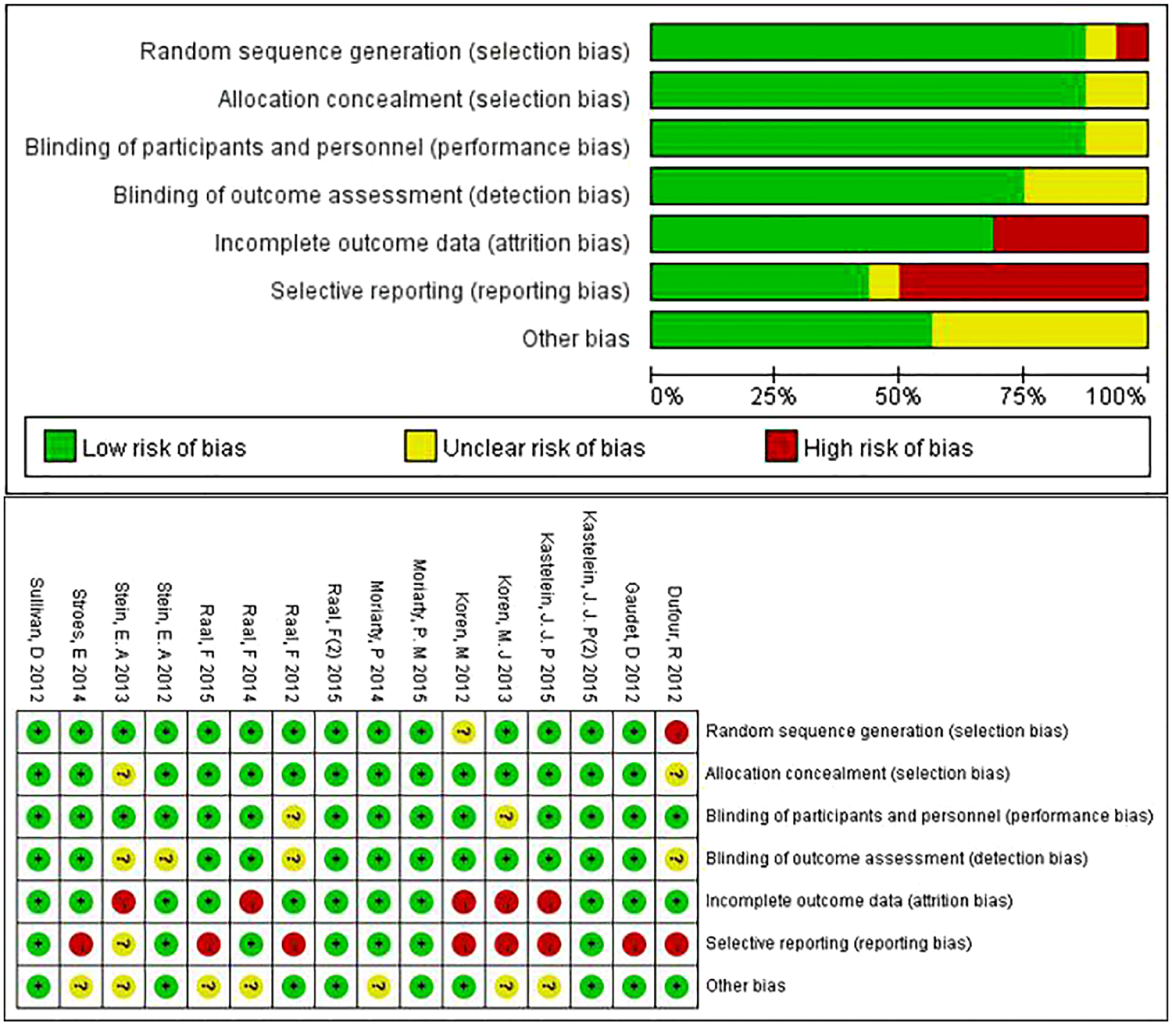
Risk-of-bias graph and summary table: review authors’ judgments about each risk-of-bias item presented as percentages across all included studies.

A total of 178 studies were searched in our systematic literature, with 43 duplicate publications. 106 studies were unable to meet the inclusion criteria excluded; 14 studies were further ruled out, where 6 studies were articles, 2 studies had no control groups, 1 study was not RCT, 1 was open label trial and 3 were not meta-analysis with quantitative synthesis. (Figure 1) As a result, 15 studies encompassing a total of 4,288 patients were selected^26–40^. Among them, 7 trials used evolocumab (AMG 145) and 8 studies with alirocumab (SAR236553/REGN727) treatment. Baseline characteristics were detailed giving the substantially similar basic values between PCSK9-mAbs and controls. Mean age of the subjects ranged from 31 to 65 years old. All trials were published between 2012 and 2015, followed up for 8 to 78 weeks, with a low risk of bias (Table 1 and Figure 2).

**Table 1:** Baseline Characteristics of Trials Included in Meta-Analysis.

Author, Year	Design	Diagnosis	Control	Drug Regimen	Duration	Number	Mean Age (y)
Raal, F, 2012	M, R, DB, PC, MD	HeFH	Placebo	E: 350 mg SC q4w/E:420 mg SC q4w/placebo SC q4w	12 weeks	168	50 (13)
Sullivan, D, 2012	M, R, DB, PC, MD	statin-intolerant patients	Placebo	E: 280 mg SC q4w/E: 350 mg SC q4w/E: 420 mg SC q4w/E: 420 mg + ezetimibe: 10 mg SC q4w/ezetimibe: 10 mg + placebo SC q4w.	12 weeks	236	62
Stein, E. A, 2013	M, R, DB, PC	statin-intolerant patients	SOC	E: 420 mg SC q4w + SOC/SOC	54 weeks	157	NR
Raal, F, 2014	M, R, DB, PC	HoFH, >12 years old	Placebo	E: 420 mg SC q4w/placebo: SC q4w	12 weeks	51	30.9(12.8)
Stroes, E, 2014	M, R, DB, PC, MD, EC	statin-intolerant patients	Placebo, Ezetimibe	E: 140 mgSC q2w + daily oral placebo/E:420 mg SC q4w + daily oral placebo/subcutaneous placebo q2w + daily oral ezetimibe 10 mg/subcutaneous placebo q4w + daily oral ezetimibe 10 mg	12 weeks	307	62(10)
Raal, F, 2015	M, R, DB, PC	HoFH, >12 years old	Placebo	E: 420 mg SC q4w/placebo: SC q4w	12 weeks	50	31(13)
Raal, F, 2015	M, R, DB, PC, MD	HeFH	Placebo	E: 140 mg SC q2w/E: 420 mg SC q4w/placebo: SC q2w/placebo: SC q4w	12 weeks	331	51.9
Dufour, R, 2012	M, R, DB, PC	HeFH + nonFH	Placebo	A: 50–300 mg SC either q2w or q4w/placebo SC either q2w or q4w(background statin or statin + ezetimibe 9 mg)	12 weeks	352	65
Gaudet, D, 2012	M, R, DB, PC, AD	HeFH + nonFH	Placebo	A:50–300 mg SC either q2w or q4w/placebo SC either q2w or q4w(background statin or statin + ezetimibe 10 mg)	12 weeks	352	65
Koren, M, 2012	M, R, DB, PC	HeFH + nonFH	Placebo	A: any other regimen/A: 150 mg SC q2w/placebo SC q2w	8–12 weeks	352	NR
Stein, E. A, 2012	M, R, DB, PC, MD	HeFH + nonFH	Placebo	A: 0.3 mg/kg or placebo Intravenous; A: 50, 100, or 150 mg SC on days 1, 29, and 43	148 days	133	45
Koren, M. J, 2013	M, R, DB, PC, AD	HeFH + nonFH	Placebo	A: 150 mg SC q2w/a50–300 mg, SC either q2W or q4W/placebo SC either q2W or q4W	8–12 weeks	351	NR
Moriarty, P, 2014	M, R, DB, PC	HeFH + nonFH, LDL-C > 2.6 mmol/L	Placebo	A: 150 mg SC q2w/placebo SC q2W	8–12 weeks	352	65
Kastelein, J. J. P, 2015	M, R, DB, PC, MD	HeFH	Placebo	A: 75 mg/placebo SC q2w; A: 150 mg SC q2w/placebo SC q2w	78weeks	735	53
Moriarty, P. M., 2015	M, R, DB, PC, MD, EC	statin-intolerant patients	Placebo, ezetimibe, atorvastatin	A: 75 mg SC q2w(+oral placebo)/ezetimibe 10 mg/d (+SC placebo q2w)/atorvastatin 20 mg/d (+SC placebo Q2W); A: 150 mg SC q2w(+oral placebo)/ezetimibe 10 mg/d (+SC placebo q2w)/atorvastatin 20 mg/d (+SC placebo Q2W)	24weeks	361	63

A = Alirocumab/REGN727; AD = ascending dose; DB = double blind; DR = dose ranging; E = Evolocumab/AMG145; EC = ezetimibe control; HeFH = heterozygous familial hypercholesterolemia; HoFH = homozygous familial hypercholesterolemia; nonFH = not known familial hypercholesterolemia; IV = intravenous; LDL-C = low-density lipoprotein cholesterol; M = multicenter; MD = multiple dose; NR = not reported; PC = placebo control; PG = parallel group; q2w = every 2 weeks; q4w = every 4 weeks; qw = once weekly; R = randomized; S = single center; SC = subcutaneous; SOC = standard of care.

### Quantitative synthesis and heterogeneity of data

**Figure 3:**
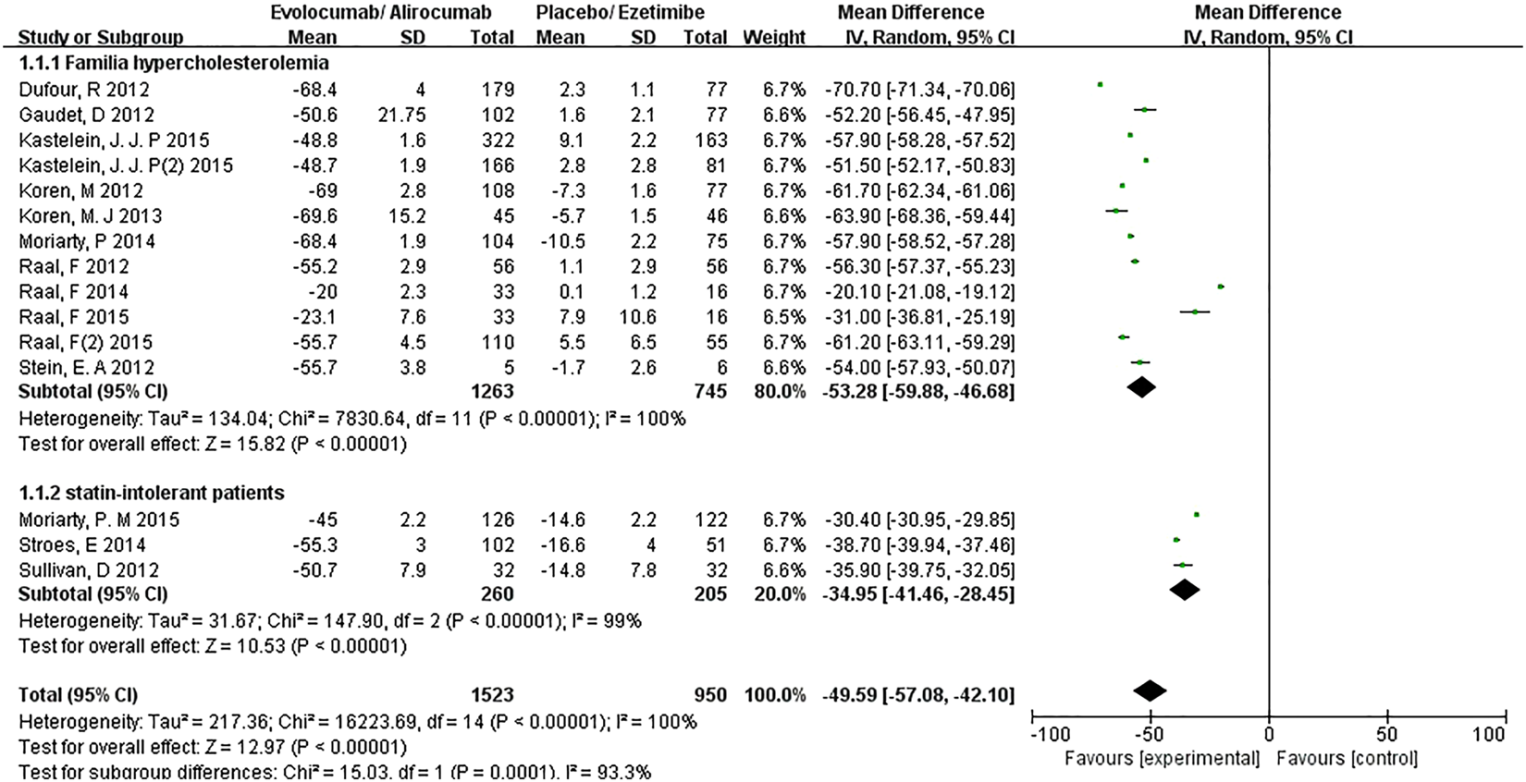
Forest plot depicting the efficacy outcomes of PCSK9 monoclonal antibody on familial hypercholesterolemia and statin-intolerant patients.

According to the efficacy outcomes of PCSK9-mAbs on FH and statin-intolerant patients, the LDL-C level dropped by the greatest margin, −49.59%, 95% CI: −57.08 to −42.10% versus control groups. There was significant heterogeneity in the comparisons (I^2^ = 100%). The reduction of LDL-C level in FH (−53.28%, 95% CI: −59.88 to −46.68%) was even greater than that in statin-intolerant patients (−34.95%, 95% CI: −41.46 to −28.45%). **(**Figure 3).

### Efficacy outcomes of PCSK9-mAbs

**Figure 4:**
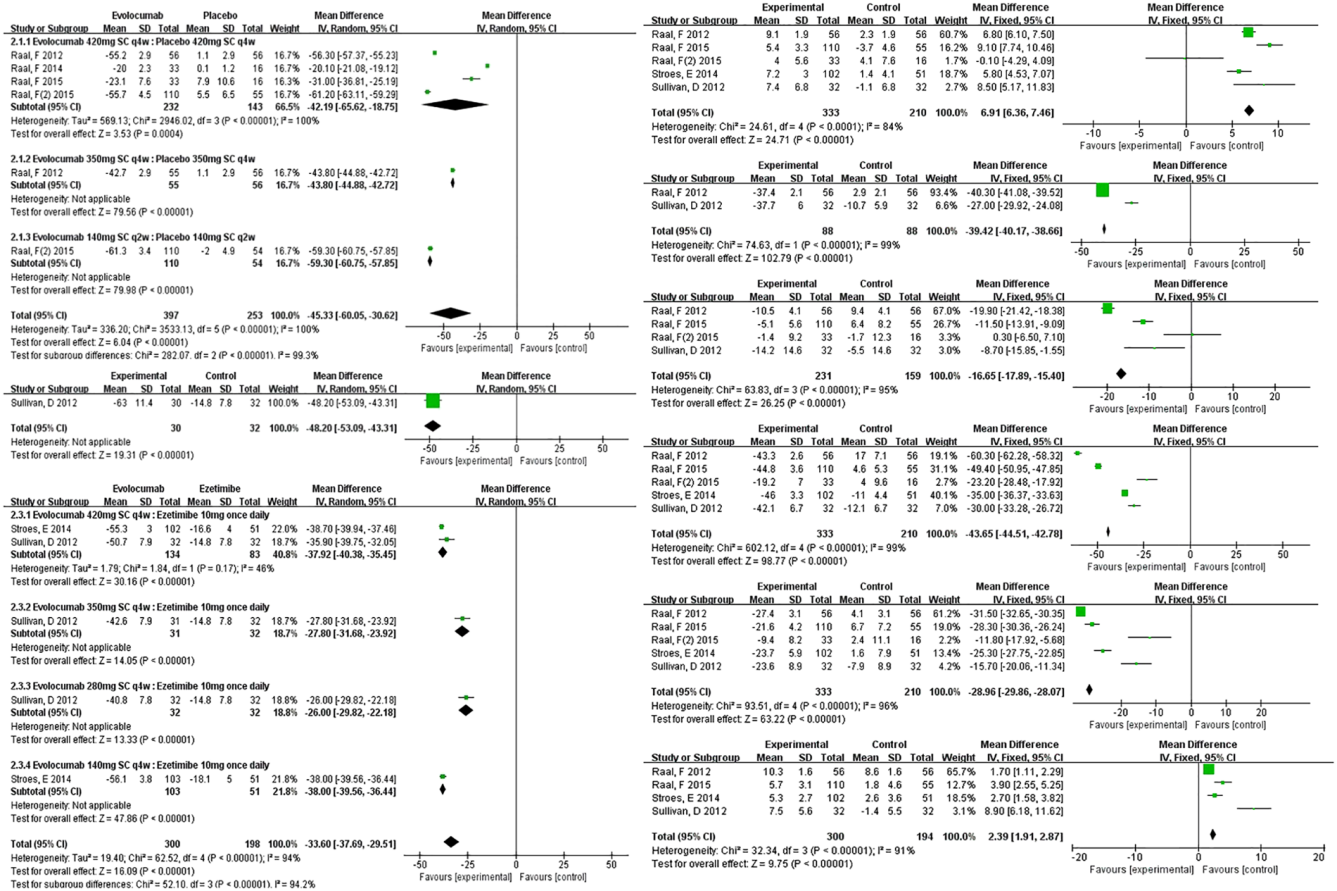
Forest plots depicting the effect of Evelocumab for subgroup analysis after grouping by methods of treatment. (**a**) on LDL-C; (**b**) on HDL; (**c**) on TC; (**d**) on TG; (**e**) on Apo-B; (**f**) on Lp(a); (**g**) on Apo-A1.

As lipid outcomes of evolocumab, significant reduction of LDL-C level was achieved in all dosages of evolocumab (mean reduction: −51.75%, 95% CI: −53.58 to 49.92%, I^2^ = 92.6%), with the greatest decrease in biweekly 140 mg evolocumab (−59.30%, 95% CI: −60.75 to −57.85%), monthly 350 mg evolocumab (−43.80%, 95% CI: −44.88 to −42.72%), monthly 420 mg evolocumab (−42.19%, 95% CI: −65.62 to −18.75%) versus placebo. There was significant heterogeneity in the comparisons (I^2^ = 99.3%). The effect might be dose-dependent and biweekly 140 mg evolocumab tends to be the optimal administration. Compared with ezetimibe or atrovastatin controls, obvious LDL-C reduction also occurred in all dosages of evolocumab, −30.4% (95% CI: −30.95 to −29.85%) versus ezetimibe and −48.9% (95% CI: −51.27 to −46.53%), −55.90% (95% CI: −58.76 to −53.04%) versus atrovastatin controls respectively. No significant heterogeneity was detected in the comparisons (I^2^ = 0%), demonstrating great similar but less remarkable outcome s compared with those versus controls. (Figure 4a).

In addition, HDL-C level obviously increaded by 6.91% (95% CI: 6.36 to 7.46%, I^2^ = 84%) and Apo-A1 level by 2.39% (95% CI: 1.91 to 2.87%, I^2^ = 91%) versus placebo, while significant reductions of TC, TG, Lp(a) and Apo-B were generated by monthly 420 mg evolocumab treatment, which were −39.42% (95% CI: −40.17 to −38.66%), −16.65% (95% CI: −17.89 to −15.40%), −28.96% (95% CI: −29.86 to −28.07%) and −43.65% (95% CI: −44.51 to −42.78%) respectively, with levels of heterogeneity (I^2^ = 99%, 95%, 99% and 96%). (Figure 4b–g).

**Figure 5:**
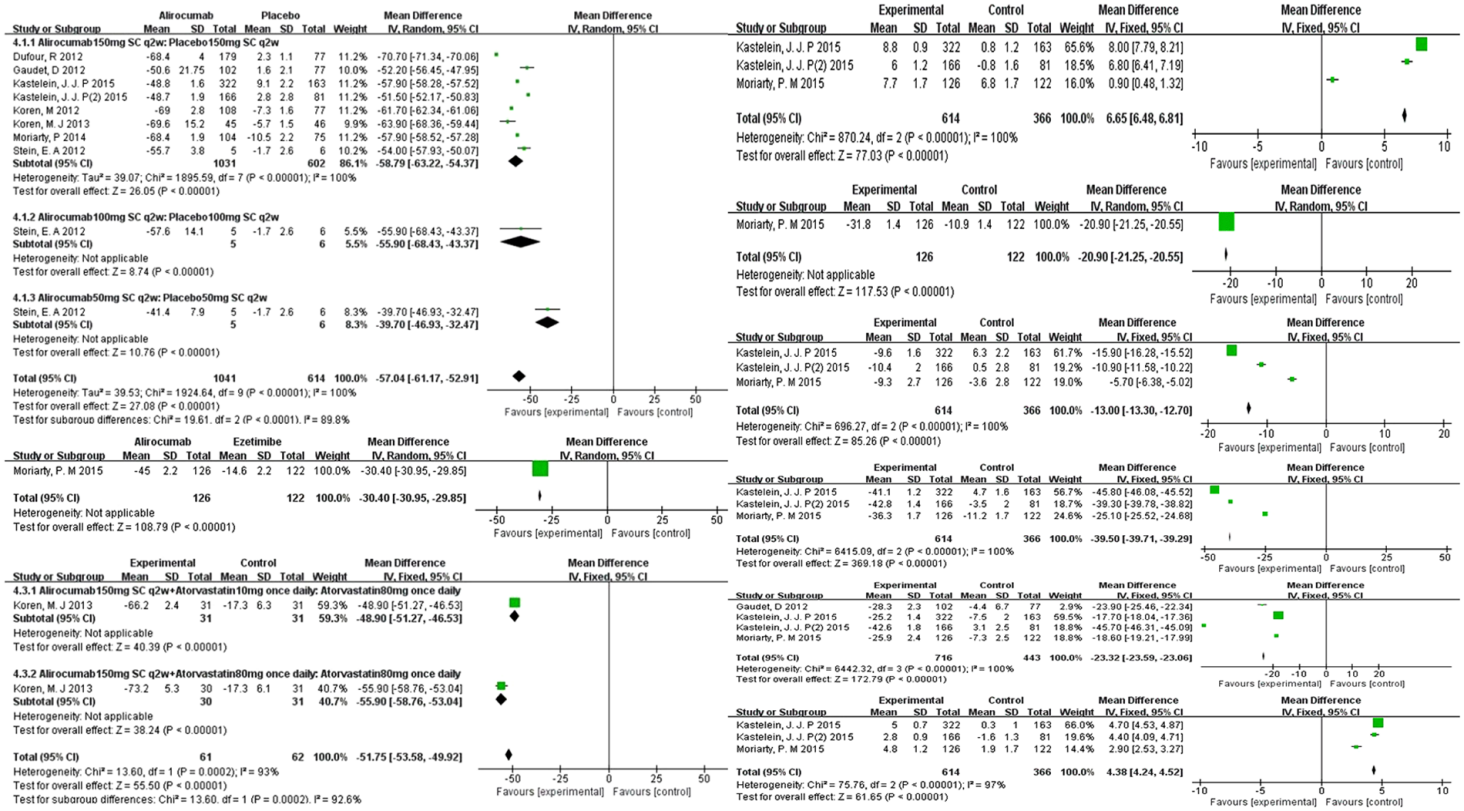
Forest plots depicting the effect of Alirocumab for subgroup analysis after grouping by methods of treatment. (**a**) on LDL-C; (**b**) on HDL; (**c**) on TC; (**d**) on TG; (**e**) on Apo-B; (**f**) on Lp(a); (**g**) on Apo-A1.

Concluding lipid outcomes of alirocumab, LDL-C level was obviously decreased by all dosages of alirocumab (mean reduction: −57.04%, 95% CI: −61.17 to −52.91%), with significant heterogeneity in the comparison (I^2^ = 100%). Significant reductions were achieved by biweekly 150 mg alirocumab (58.79%, 95% CI: −63.22 to −54.37%), biweekly 100 mg alirocumab (−55.90%, 95% CI: −68.43 to −43.37%) and biweekly 50 mg alirocumab (−39.70%, 95% CI: −46.93 to −32.47%) versus placebo. With the same frequency of administration, greater reduction was achieved by biweekly 150 mg than 100 or 50 mg alirocumab, adding more evidences to dose dependent effect. Compared with ezetimibe or atrovastatin controls, significant reductions of LDL-C also occurred: −30.40% (95% CI: −30.95 to −29.85%) in alirocumab versus ezetimibe; −48.90% (95% CI: −51.27 to −46.53%) in alirocumab versus placebo both under atorvastatin 10 mg once daily; −55.90% (95% CI: −58.76 to −53.04%) in alirocumab versus placebo both under atorvastatin 80 mg once daily, with no significant heterogeneity. Few studies showed largely changes of LDL-C level versus ezetimibe or atorvastatin controls, demonstrating clearly similar but less remarkable results. (Figure 5a).

Biweekly 150 mg alirocumab treatment significantly elevated HDL-C level by 6.65% (95% CI: 6.48 to 6.81%, I^2^ = 100%) and Apo-A1 level by 4.38% (95% CI: 4.24 to 4.52%, I^2^ = 97%) versus placebo. Substantial reductions of TC, TG, Lp(a), Apo-B were respectively achieved by −20.90% (95% CI: −21.25 to −20.55%, I^2^ = 0), −13.00% (95% CI: −13.30 to −12.70%, I^2^ = 100%), −23.32% (95% CI: −23.59 to −23.06%) and −39.50% (95% CI: −39.71 to −39.29%, I^2^ = 100%). (Figure 5b–g).

### Safety outcomes of PCSK9-mAbs

**Figure 6:**
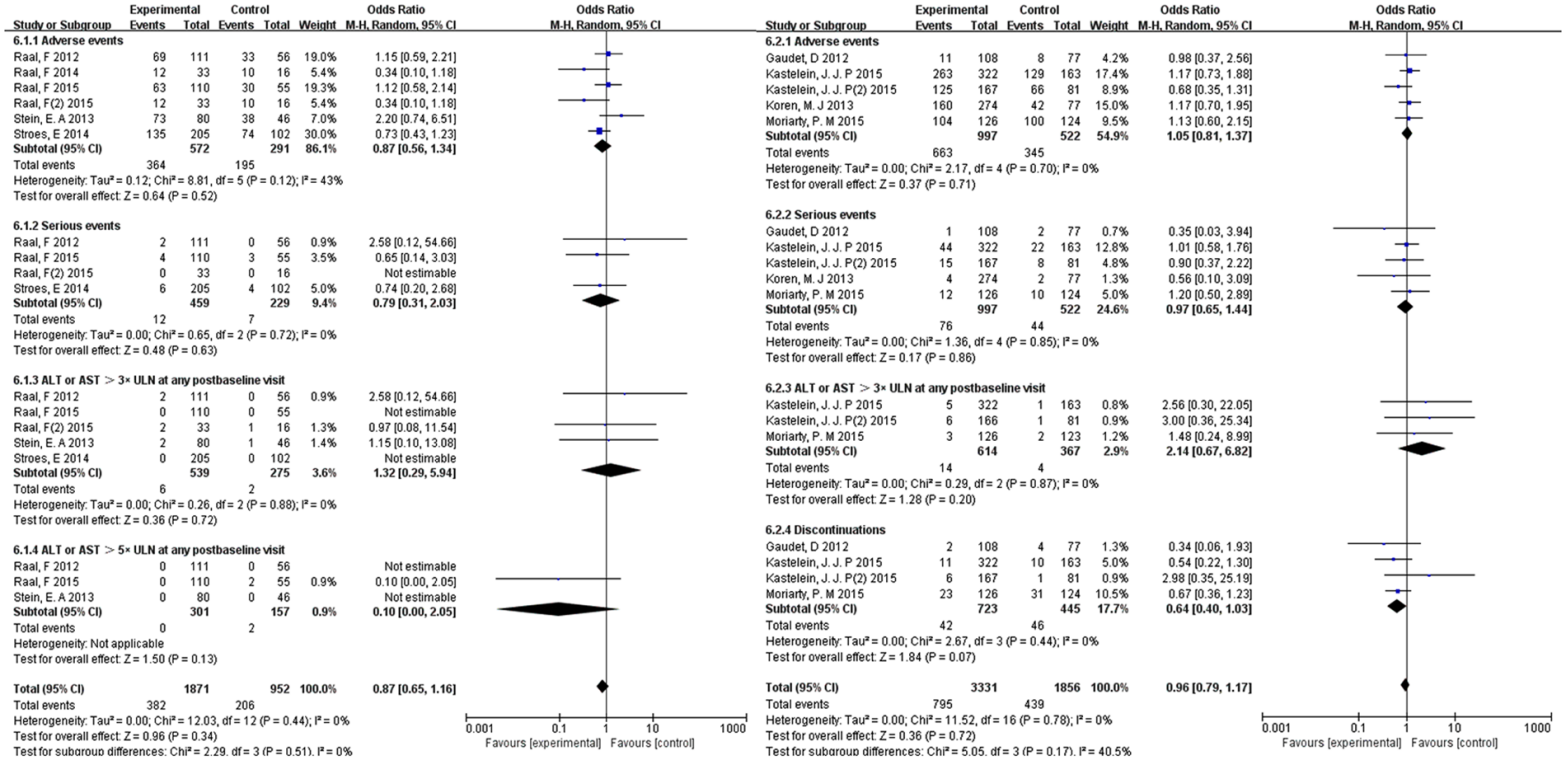
Forest plot depicting the adverse event rates following (**a**) evolocumab and (**b**) alirocumab therapies, compared with placebo or ezetimibe controls on adverse events, serious events and ALT or AST rates.

Safety endpoints covering the common adverse events, serious events and laboratory adverse events were compared between the PCSK9-mAbs and control groups. The pooled estimation of evolocumab showed the overall incidence of common adverse events (RR: 0.87, 95% CI:0.56 to 1.34, I^2^ = 43%) and serious events(RR: 0.79, 95% CI: 0.31 to 2.03, I^2^ = 0%) versus placebo, which implied no obvious difference. No significant heterogeneity was found in hepatotoxicity risk analysis of evolocumab with levels of alanine aminotransferase (ALT) or aspartate aminotransferase (AST) no greater than three times the upper limit of normal (ULN) by RR: 1.32, 95% CI: 0.29 to 5.94, I^2^ = 0%. (Figure 6a) The alirocumab treatment implied no significant difference versus placebo, with the common adverse events by RR: 1.05, 95% CI: 0.81 to 1.37, serious events by RR: 0.97, 95% CI: 0.65 to 1.44 versus placebo. No significant heterogeneity was found in the analysis of abnormal liver function risk (AST/ALT ≥ 3 × ULN) in patients by RR: 2.14, 95% CI: 0.67 to 6.82). (Figure 6b) We have included an additional table for pre-specified safety end point which describes the safety events of interest, common adverse events and laboratory results of PCSK9-mAbs. No statistical differences between PCSK9-mAbs and control group except any and paraesthesia events, which were positive in the PCSK9-mAbs group. χ^2^ statistics was to assess the magnitude of heterogeneity and P value < 0.05 was considered to be statistically significant. (Table 2).

**Table 2:** Pre-specified Safety End Points.

Pre-specified Safety End Points	Control group		PCSK9-mAbs group		χ^2^	P value
	No. of patients/objects	Rate(%)	No. of patients/objects	Rate(%)		
**Safety events of interest**						
Deaths	0/718	0	6/1381	0.43	3.115	0.101
Coronary artery disease	0/188	0	3/283	1.06	1.985	0.281
Ischemia-driven coronary revascularization procedure	1/159	0.63	8/197	4.06	4.014	0.084
CHF requiring hospitalization	0/35	0	1/72	1.39	0.484	1
Adjudicated cardiovascular events	5/368	1.36	14/615	2.28	0.986	0.473
**Common adverse events**						
Any	632/10593	5.97	988/19070	5.18	7.276	0.007
Serious	53/779	6.80	102/1454	7.02	0.031	0.931
Development/worsening of diabetes	7/279	2.51	11/561	1.96	0.255	0.619
Hepatic disorders	3/35	8.57	4/72	5.56	0.304	0.684
General allergic events	23/279	8.24	52/561	9.27	0.202	0.702
Ophthalmologic disorders	5/279	1.79	7/561	1.25	0.38	0.547
Discontinuation of investigational product	64/803	7.97	83/1440	5.76	3.573	0.063
Upper respiratory tract infection	120/2137	5.62	247/4343	5.69	0.012	0.955
Neurological disorders	67/1168	5.74	110/2405	4.57	2.036	0.164
Neurocognitive disorders	3/369	0.81	2/742	0.27	1.607	0.341
Digestive tract disorders	30/733	4.09	53/1532	3.46	0.522	0.475
Injection site reactions	49/820	5.98	102/1493	6.83	0.559	0.483
Muscle-related disorders	251/3378	7.43	306/4730	6.47	2.476	0.12
Paraesthesia	9/204	4.41	2/410	0.49	11.358	0.002
Contusion	1/109	0.92	9/220	4.09	2.369	0.176
**Laboratory results**						
ALT, AST, or both ≥3 × ULN	5/763	0.66	19/1408	1.35	2.138	0.196
CK > 3 × ULNat any post-baseline shift	27/2209	1.22	47/3298	1.43	0.4	0.553
hsCRP, maximum post-baseline shift, (%)1 to 3 mg/dL	0/53	0	2/108	1.85	0.976	1
hsCRP, maximum post-baseline shift, (%) >3 mg/dL	0/53	0	1/108	0.93	0.489	1
Total bilirubin level, >2.5 mg/dL	0/40	0	1/32	3.13	1.229	0.452

No statistical differences between PCSK9-mAbs and control group except any and paraesthesia events, which were positive in the PCSK9-mAbs group.

PCSK9-mAbs = PCSK9-monoclonal antibodies; CHF = congestive heart failure; Any = any of the common adverse events, ALT = alanine aminotransferase; AST = aspartate aminotransferase; ULN = upper limit of normal; CK = creatinine kinase ; hsCRP = hypersensitive C reactive protein.

## Discussion

As far as we know, this is the first meta-analysis using sufficient clinical outcomes comprehensively to define the applicable targets of PCSK9-mAbs—FH and statin-intolerant patients. In the present analysis, a total of 15 studies encompassing 4,288 patients with FH and statin-intolerence were included. The main point addressed is whether PCSK9-mAb treatment have the ability to improve the lipid levels of FH and statin-intolerant patients with satisfactory safety and tolerability.

**Figure 7:**
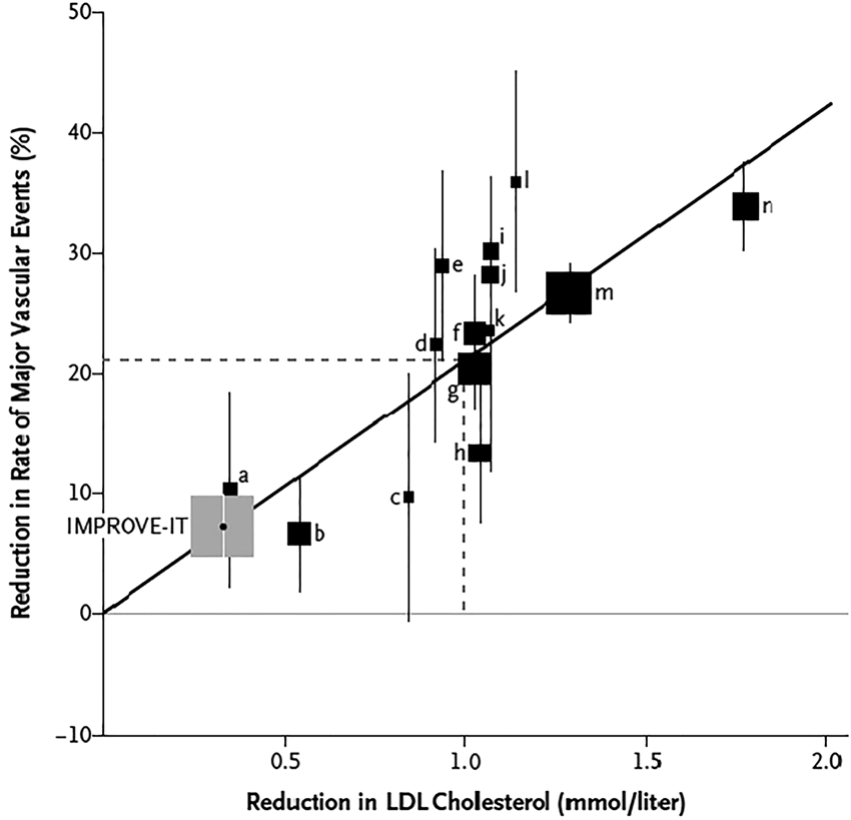
Plot of the IMPROVE-IT Trial Data and Statin Trials for Change in LDL-C versus Clinical Benefit.

At present, additional on-going large-sized multicenter randomized studies were not included in our study, such as the Phase III FOURIER, GLAGOV and GAUSS-3 trial^41–43^, which are intended to determine if PCSK9-mAbs can help to not only reduce LDL-C level obviously, but also improve cardiovascular related events such as myocardial infarction(MI), and the rates of morbidity and mortality. Nevertheless, the concept of LDL-C reduction, as a surrogate for prevention of long-term cardiovascular outcomes in high-risk patients with acute coronary syndromes, was provided by the recently published IMPROVE-IT study with the strongest clinical trial evidence. The more reduction of LDL-C, the rate of major vascular events decreases^44^. (Figure 7) Given the consistent effects on LDL-C reduction, PCSK9-mAbs appears to be a promising approach to cut down the risk for cardiovascular events in the patients selected.

In a broader sense, LDL particles, non-HDL-C, the Apo-B/Apo-A1 ratio, and Lp(a) may be the better replacement markers for cardiovascular mortality in the foreseeable future, compared with LDL-C levels^45–48^. Overall, results of the study are suggestive of therapeutic efficacy of PCSK9-mAbs in curtailing the major risk factors (including LDL-C, TC, TG, Lp(a) and Apo-B) of CVD, and increasing HDL-C and Apo-A1 by detecting both evelocumab and alirocumab. It has been previously suggested that Apo-B/Apo-A1 ratio is a better risk indicator for CVD and MI than the level of lipids^49^. In our study, Apo-B decreased while Apo-A1 elevated in the PCSK9-mAb treatment group. The decrease in Apo-B/Apo-A1 ratio obviously indicates that PCSK9-mAb therapy greatly lowers the primary risk factors of heart disease. We also found that the reduction of LDL-C in FH patients (−53.28%, 95% CI: −59.88 to −46.68%) was greater than that in statin-intolerant patients (−34.95%, 95% CI: −41.46 to −28.45%). In terms of safety, PCSK9-mAbs may lead to many events, none of which is life-threatening; the serious events do not increase compared with the control group; more large RCTs are needed to further confirm the safety.

Recently, there have been four meta-analyses comparing the effects of PCSK9-mAbs over placebo controls, with or without considering statin therapy^50–53^. In these studies, AMG145/Evolocumab, REGN727/SAR236553/Alirocumab were primarily included. However, novel clinical studies are being done to evaluate the target of LDL-C, such as RN316/bococizumab, RG7652, LY 3015014, ALN-PCSSC, which has been genetically validated to lower the cardiovascular risk.

The mechanism of improving the survival of patients treated with PCSK9mAb remains obscure and its role in the observed survival benefit, for its encouraging result, should be interpreted with caution. The efficacy of these agents in reducing lipid levels (particularly LDL-C) and rates of MI due to plaque stabilization is well-evaluated. Moreover, the lower frequency of PCSK9-mAbs intake may lead to slighter hepatotoxicity. However, subcutaneous injection of PCSK9-mAb makes it an alternative for drug adherence. What’s more, studies reveal that LDL-R can act as the entry point for hepatitis C virus, which may increase the risk of infection^54^. Finally, it still remains unknown whether the reduced LDL-C by PCSK9-mAbs can improve the long-term clinical outcomes^55^.

Several limitations should be taken into consideration. First, a few studies have only been reported in abstracts or presented at meetings, leading to added detection bias. Besides, significant heterogeneities were observed in most of the reported outcomes, but we failed to reveal the heterogeneities by dividing subgroups or sensitivities methods. Some large multicenter RCTs are still under investigation. Therefore, caution should be taken in interpreting the results of the meta-analysis when combining the heterogeneous data sets. Despite these limitations, our meta-analysis covers the most applicable targets of PCSK9-mAbs—FH and statin-intolerant patients, and their clinical outcomes are sufficient enough to compensate our clinical guidelines. Hopefully, more RCTs could be carried out to provide more evidences about its long-term therapeutic efficacy, safety, and clinical outcomes.

## Methods

### Study searching

In order to identify appropriate RCTs, a comprehensive literature search was performed throughout PubMed, EMBASE and The Cochrane Library databases, with the following terms and key words: evolocumab, AMG 145, alirocumab, SAR236553, REGN727x, PCSK9 monoclonal antibodies, FH, statin intolerant and randomized controlled trial from it’s inception in 2012 to May 26, 2016. All the selected studies, restricted to English, were initially screened for relevance at the abstract level.

### Study selection

The inclusion criteria are as follows: (1) RCTs; (2) population: FH, statin intolerant patients; (3) safety and efficacy outcomes of PCSK9-mAbs; (4) mean differences (MDs) with 95% corresponding confidence interval (95% CI) and so on. Studies that were not randomized, special-population-targeted or designed to test PCSK9-mAbs with limited number of trials (such as bococizumab, RG7652, LY3015014 and ALN-PCSSC) were excluded.

### Outcomes

The primary efficacy endpoints included parameter changes following PCSK9-mAbs treatment from baseline: (1) LDL-C reduction; (2) other lipid profile changes. Therapeutic safety was evaluated by rates of the common adverse events, serious events and laboratory adverse events respectively.

### Data collection

Two investigators (LJQ and YG) performed eligibility assessment with a standardized data extraction individually and another reviewer (YMZ) checked the data. Basic information was extracted as follows: study/author, year, design, diagnosis, control, drug regimen, duration, patient number, mean age (y) at baseline. As a precedence, we extracted the corresponding mean differences, 95% CI from baseline for each lipid items, such as LDL-C, HDL-C, TC, TG, Apo-B and Apo-A1 levels, before and after PCSK9-mAbs treatment as the primary outcomes. Safety endpoints covering the common adverse events, serious events and laboratory adverse events were compared between the treatment and control groups. In addition, the common adverse events included injection site reaction (e.g. generalized pruritis, hypersensitive reaction, erythema, rash, swelling, discoloration, or pain), nasopharyngitis, upper respiratory tract infections, influenza, cough, nausea, myalgia, myositis, headache, diarrhea, fatigue, abnormal pain, rectal bleeding, dehydration, arthralgia, and back pain. The serious events, fatal and life threatening, require hospital admission or prolonged stay in the hospital for the possibility of persistent or significant disability. The laboratory adverse events evaluated hepatotoxicity with ALT or AST levels ≥3 × ULN.

### Quality assessment

Two reviewers (LJQ and YG) assessed the risk of bias with the Cochrane Collaboration’s tool (Review Manager version 5.3) in the included trials. For efficacy outcomes, comparisons of LDC-C reduction were performed separately on FH and statin-intolerant patients. The changes of each lipid items stratified by forms and dosages of PCSK9-mAbs were carried out versus placebo or ezetimibe treatment. We assessed the publication bias, including the risk of selection bias, performance bias, detection bias, attrition bias, reporting bias and other bias, by using the Risk-of-bias graph and summary table.

### Statistical analysis

All analyses were conducted with Review Manager version 5.3. A fixed-effect model was selected if there was no unexplained statistical heterogeneity, otherwise, a random-effect model was used in the meta-analysis. Cochrane Q test to measure the heterogeneity among the included trials and I^2^ statistics to assess the magnitude of heterogeneity were performed separately. χ^2^ statistics was to assess the magnitude of heterogeneity and P value < 0.05 was considered to be statistically significant.

## Electronic supplementary material

Table 1

Table 2

Title page
