# Supplementary material for: Therapeutic efficacy and safety of PCSK9-monoclonal antibodies on familial hypercholesterolemia and statin-intolerant patients: A meta-analysis of 15 randomized controlled trials
Source: Sci Rep. 2017 Mar 22;7:238. doi: 10.1038/s41598-017-00316-3 (PMC5428249; doi:10.1038/s41598-017-00316-3)
Supplement: Supplementary file 1 — Table 1 [file 41598_2017_316_MOESM1_ESM.doc]

| Author, Year | Design | Diagnosis | Control | Drug Regimen | Duration | Number | Mean Age (y) |
| --- | --- | --- | --- | --- | --- | --- | --- |
| Raal, F,2012 | M, R, DB, PC,MD | HeFH | Placebo | E :350mg SC q4w /E:420mg SC q4w/ placebo SC q4w | 12 weeks | 168 | 50 [13] |
| Sullivan, D,2012 | M, R, DB, PC,MD | statin-intolerant patients | Placebo | E:280 mg SC q4w/E:350 mg SC q4w/E:420 mg SC q4w/ E:420 mg+ezetimibe:10 mg SC q4w /ezetimibe:10 mg + placebo SC q4w. | 12 weeks | 236 | 62 |
| Stein, E. A,2013 | M, R, DB, PC | statin-intolerant patients | SOC | E:420 mg SC q4w+SOC/ SOC | 54 weeks | 157 | NR |
| Raal, F,2014 | M, R, DB, PC | HoFH,>12 years old | Placebo | E :420 mg SC q4w /placebo:SC q4w | 12 weeks | 51 | 30.9(12.8) |
| Stroes, E,2014 | M, R, DB, PC,MD,EC | statin-intolerant patients | Placebo, Ezetimibe | E:140 mgSC q2w + daily oral placebo / E:420 mg SC q4w + daily oral placebo / subcutaneous placebo q2w + daily oral ezetimibe 10 mg / subcutaneous placebo q4w + daily oral ezetimibe 10 mg | 12 weeks | 307 | 62(10) |
| Raal, F,2015 | M, R, DB, PC | HoFH,>12 years old | Placebo | E :420 mg SC q4w /placebo:SC q4w | 12 weeks | 50 | 31(13) |
| Raal, F,2015 | M, R, DB, PC,MD | HeFH | Placebo | E:140mg SC q2w /E:420mg SC q4w/placebo:SC q2w/placebo:SC q4w | 12 weeks | 331 | 51.9 |
| Dufour, R,2012 | M, R, DB, PC | HeFH+nonFH | Placebo | A:50-300 mg SC either q2w or q4w /placebo SC either q2w or q4w(background statin or statin + ezetimibe 9 mg) | 12 weeks | 352 | 65 |
| Gaudet, D,2012 | M, R, DB, PC,AD | HeFH+nonFH | Placebo | A:50-300 mg SC either q2w or q4w /placebo SC either q2w or q4w(background statin or statin + ezetimibe 10 mg) | 12 weeks | 352 | 65 |
| Koren, M,2012 | M, R, DB, PC | HeFH+nonFH | Placebo | A:any other regimen/A:150mg SC q2w /placebo SC q2w | 8-12 weeks | 352 | NR |
| Stein, E. A,2012 | M, R, DB, PC,MD | HeFH+nonFH | Placebo | A:0.3 mg/kg or placebo Intravenous; A:50, 100, or 150 mg SC on days 1, 29, and 43 | 148 days | 133 | 45 |
| Koren, M. J,2013 | M, R, DB, PC,AD | HeFH+nonFH | Placebo | A:150 mg SC q2w /a50-300 mg,SC either q2W or q4W/placebo SC either q2W or q4W | 8-12 weeks | 351 | NR |
| Moriarty, P,2014 | M, R, DB, PC | HeFH+nonFH,LDL-C >2.6 mmol/L | Placebo | A:150 mg SC q2w /placebo SC q2W | 8-12 weeks | 352 | 65 |
| Kastelein, J. J. P,2015 | M, R, DB, PC,MD | HeFH | Placebo | A:75 mg/placebo SC q2w; A:150 mg SC q2w/placebo SC q2w | 78weeks | 735 | 53 |
| Moriarty, P. M.,2015 | M, R, DB, PC,MD,EC | statin-intolerant patients | Placebo,  ezetimibe,  atorvastatin | A:75 mg SC q2w(+oral placebo)/ezetimibe 10 mg/d (+SC placebo q2w)/atorvastatin 20 mg/d (+SC placebo Q2W);A:150 mg SC q2w(+oral placebo)/ezetimibe 10 mg/d (+SC placebo q2w)/atorvastatin 20 mg/d (+SC placebo Q2W) | 24weeks | 361 | 63 |

Table 1 Baseline Characteristics of Trials Included in Meta-Analysis

A indicates Alirocumab/REGN727; AD, ascending dose; DB, double blind; DR, dose ranging; E, Evolocumab/AMG145; EC, ezetimibe control; HeFH, heterozygous familial hypercholesterolemia; HoFH, homozygous familial hypercholesterolemia; nonFH, not known familial hypercholesterolemia; IV, intravenous; LDL-C, low-density lipoprotein cholesterol; M, multicenter; MD, multiple dose; NR, not reported; PC, placebo control; PG, parallel group; q2w, every 2 weeks; q4w, every 4 weeks; qw, once weekly; R, randomized; S, single-center; SC, subcutaneous; SOC, standard of care.
