# Supplementary material for: Therapeutic efficacy and safety of PCSK9-monoclonal antibodies on familial hypercholesterolemia and statin-intolerant patients: A meta-analysis of 15 randomized controlled trials
Source: Sci Rep. 2017 Mar 22;7:238. doi: 10.1038/s41598-017-00316-3 (PMC5428249; doi:10.1038/s41598-017-00316-3)
Supplement: Supplementary file 2 — Table 2 [file 41598_2017_316_MOESM2_ESM.doc]

| Pre-specified Safety End Points | | | | | |  |
| --- | --- | --- | --- | --- | --- | --- |
| Safety events of interest | Control group | | PCSK9-mAbs group | | χ2 | P value |
|  | No. of patients/objects | Rate(%) | No. of patients/objects | Rate(%) |  |  |
| Deaths | 0/718 | 0 | 6/1381 | 0.43 | 3.115 | 0.101 |
| Coronary artery disease | 0/188 | 0 | 3/283 | 1.06 | 1.985 | 0.281 |
| Ischemia-driven coronary revascularization procedure | 1/159 | 0.63 | 8/197 | 4.06 | 4.014 | 0.084 |
| CHF requiring hospitalization | 0/35 | 0 | 1/72 | 1.39 | 0.484 | 1 |
| Adjudicated cardiovascular events | 5/368 | 1.36 | 14/615 | 2.28 | 0.986 | 0.473 |

| Common adverse events | Control group | | PCSK9-mAbs group | | χ2 | P value |
| --- | --- | --- | --- | --- | --- | --- |
|  | No. of patients/objects | Rate (%) | No. of patients/objects | Rate (%) |  |  |
| Any | 632/10593 | 5.97 | 988/19070 | 5.18 | 7.276 | 0.007 |
| Serious | 53/779 | 6.80 | 102/1454 | 7.02 | 0.031 | 0.931 |
| Development/worsening of diabetes | 7/279 | 2.51 | 11/561 | 1.96 | 0.255 | 0.619 |
| Hepatic disorders | 3/35 | 8.57 | 4/72 | 5.56 | 0.304 | 0.684 |
| General allergic events | 23/279 | 8.24 | 52/561 | 9.27 | 0.202 | 0.702 |
| Ophthalmologic disorders | 5/279 | 1.79 | 7/561 | 1.25 | 0.38 | 0.547 |
| Discontinuation of investigational product | 64/803 | 7.97 | 83/1440 | 5.76 | 3.573 | 0.063 |
| Upper respiratory tract infection | 120/2137 | 5.62 | 247/4343 | 5.69 | 0.012 | 0.955 |
| Neurological disorders | 67/1168 | 5.74 | 110/2405 | 4.57 | 2.036 | 0.164 |
| Neurocognitive disorders | 3/369 | 0.81 | 2/742 | 0.27 | 1.607 | 0.341 |
| Digestive tract disorders | 30/733 | 4.09 | 53/1532 | 3.46 | 0.522 | 0.475 |
| Injection site reactions | 49/820 | 5.98 | 102/1493 | 6.83 | 0.559 | 0.483 |
| Muscle-related disorders | 251/3378 | 7.43 | 306/4730 | 6.47 | 2.476 | 0.12 |
| Paraesthesia | 9/204 | 4.41 | 2/410 | 0.49 | 11.358 | 0.002 |
| Contusion | 1/109 | 0.92 | 9/220 | 4.09 | 2.369 | 0.176 |

| Laboratory results | Control group | | PCSK9-mAbs group | | χ2 | P value |
| --- | --- | --- | --- | --- | --- | --- |
|  | No. of patients/objects | Rate (%) | No. of patients/objects | Rate (%) |  |  |
| ALT, AST, or both ≥3× ULN | 5/763 | 0.66 | 19/1408 | 1.35 | 2.138 | 0.196 |
| CK>3× ULN at any post-baseline shift | 27/2209 | 1.22 | 47/3298 | 1.43 | 0.4 | 0.553 |
| hsCRP, maximum post-baseline shift, (%)1 to 3 mg/dL | 0/53 | 0 | 2/108 | 1.85 | 0.976 | 1 |
| hsCRP, maximum post-baseline shift, (%) >3 mg/dL | 0/53 | 0 | 1/108 | 0.93 | 0.489 | 1 |
| Total bilirubin level, >2.5 mg/dL | 0/40 | 0 | 1/32 | 3.13 | 1.229 | 0.452 |

Table 2 Pre-specified Safety End Points

No statistical differences between PCSK9-mAbs and control group except any and paraesthesia events, which were positive in the PCSK9-mAbs group. χ2 statistics was to assess the magnitude of heterogeneity and a 2-sided P value <0.05 was considered to be statistically significant.

PCSK9-mAbs = PCSK9-monoclonal antibodies; CHF= congestive heart failure; Any = any of the common adverse events, ALT= alanine aminotransferase ; AST = aspartate aminotransferase; ULN = upper limit of normal; CK = creatinine kinase ; hsCRP = hypersensitive C reactive protein.
