# Supplementary material for: Therapeutic efficacy and safety of PCSK9-monoclonal antibodies on familial hypercholesterolemia and statin-intolerant patients: A meta-analysis of 15 randomized controlled trials
Source: Sci Rep. 2017 Mar 22;7:238. doi: 10.1038/s41598-017-00316-3 (PMC5428249; doi:10.1038/s41598-017-00316-3)
Supplement: Supplementary file 3 — Title page [file 41598_2017_316_MOESM3_ESM.doc]

Title page

**Title:** Therapeutic efficacy and safety of PCSK9-monoclonal antibodies on familial hypercholesterolemia and statin-intolerant patients: A meta-analysis of 15 randomized controlled trials

**Author list:** LiJun Qian^1^, Yao Gao^1^, YanMei Zhang^1^, MingChu^1^, Jing Yao^2^, Di Xu^1^

1 Department of Geriatrics,2 Department of Cardiology, First Affiliated Hospital of Nanjing Medical University, Nanjing 210029, China.
